# Dissociating prefrontal contribution to reactive and proactive aggression: a transcranial direct current stimulation study

**DOI:** 10.1007/s00406-025-02087-9

**Published:** 2025-08-21

**Authors:** Chiara Gramegna, Barbara Barbieri, Nadia Bolognini

**Affiliations:** 1 PhD Program in Neuroscience, School of Medicine and Surgery, University of Milano-Bicocca, Piazza dell’Ateneo Nuovo 1, Milan, 20126 Italy; 2 Department of Psychology, University of Milano-Bicocca, Milan, Italy; 3 Neuropsychological Laboratory, Department of Neurorehabilitation Sciences, IRCCS Istituto Auxologico Italiano, Milan, Italy

**Keywords:** Reactive aggression, Proactive aggression, Sex, Transcranial direct current stimulation, Dorsolateral prefrontal cortex

## Abstract

Increasing evidence suggests that the dorsolateral prefrontal cortex (dlPFC) plays a crucial role in aggression and that it may be possible to modulate this behavior using transcranial direct current stimulation (tDCS). Nevertheless, no previous study has specifically examined the differential effects of bilateral tDCS on reactive and proactive aggression, making the present research the first to explore this distinction using neuromodulation. With this aim, we examined the effect of bi-hemispheric prefrontal tDCS in 30 healthy adults using a double-blind, sham-controlled design. All participants received three types of stimulation over the dlPFC: right anodal/left cathodal, right cathodal/left anodal, and sham tDCS. During the stimulation, participants underwent a modified version of the Taylor Aggression Paradigm, which included two different tasks: one for measuring proactive aggression (i.e., pTAP) and one for reactive aggression (i.e., rTAP). They were also given self-report questionnaires measuring individual levels of aggression, impulsivity, and empathy to test whether these constructs were associated with aggressive responses at the pTAP and rTAP. Results showed increased proactive aggression in males with both active montages, while reactive aggression increased only with right cathodal/left anodal tDCS. Females exhibited increased proactive aggression during right cathodal/left anodal tDCS, but decreased reactive aggression during right anodal/left cathodal stimulation. These findings suggest sex-dependent modulation of aggression via dlPFC stimulation. The relevance of these results extends beyond healthy individuals, as dlPFC dysfunction is a common feature in several psychiatric disorders associated with aggressive behavior. Understanding how tDCS modulates these distinct forms of aggression in healthy populations can inform future research on its therapeutic potential within clinical settings.

## Introduction

Aggressiveness can be defined as a behavioral tendency to inflict physical or psychological harm on another individual [1]. However, the term is polysemous, which can be explained in two ways. First of all, it refers to the tendency to engage in hostile behavior internally motivated and linked to certain personality traits [2]. Secondly, it signifies an overt aggressive action with the intention of inflicting a violent and deceitful attack on another individual. Aggressive behavior manifested by the human population is defined as currently adaptive or resulting from adaptive strategies [3, 4]. Nevertheless, in individuals with psychiatric and/or neurological disorders, as well as in convicts and forensic psychiatric patients [5–7], overt aggression represents a significant issue, as it does not only result in the commission of criminal acts, but also precludes the possibility of providing treatment to patients and facilitating their reintegration into the community. This, in turn, gives rise to considerable costs to society [8]. The results from a recent review [9] show that the prevalence of aggressive behavior within psychiatric wards varies between 8% and 76% and that perpetrators of aggression fall into psychotic or bipolar disorders, substance use and those with a history of aggressive behavior. Furthermore, criminal behavior is common to both borderline and antisocial personality disorder patients; however, it is more frequent in the latter [8]. In patients affected by borderline personality disorder, crimes are often impulsive and explosive, whereas in antisocial subjects they tend to be characterized by instrumental forms of aggression [10]. Consequently, it becomes vital to gain an understanding of the neurobiological substrates underlying aggressive behavior, so as to propose potential supportive interventions in clinical settings, which may deal with overly aggressive patients as well as violent offenders [11].

The dualism highlighted in the very term “aggression” is also found at the basis of various theoretical models, which indicate a distinction between reactive and proactive aggressive behavior [12–15]. Nevertheless, intrinsic motivation constitutes the distinguishing feature between proactive and reactive aggression. With respect to the latter, aggressive behavior occurring in response to provocation is not goal-driven, but it arises as a response to external or internal stimuli, such as threats or frustrating events in which the main purpose is to remove the provocative stimulus. It is associated with a sudden activation of the sympathetic system followed by dysregulation of limbic-prefrontal connectivity [16–18], hence it entails a diminished activation of inhibitory functions and self-control, accompanied by an augmented proclivity towards impulsivity [18, 19]. Indeed, it is described as reactive, emotional, or “‘hot”. In contrast, proactive or “cold-blooded” aggression is characterized by a premeditated and targeted attack with the purpose of obtaining an internal or external reward. Proactive aggression is associated with low emotional activation [12, 20, 21] and its use is instrumental, manifesting when there is a high probability of achieving the desired outcome at a minimal cost [13, 22–24].

A number of factors contribute to the occurrence of aggression. These include personality traits and genetic predisposition, as well as situational factors such as the individual response to provocation and frustration [25]. It has been shown that reactive behavior results from an impulsive and defensive response that is expressed in an aversive manner to provocation [12, 20, 26]. Individuals who exhibit reactive aggression tend to display a personality profile that is characterised by impulsivity and high levels of hostility. Indeed, specific personality traits may predispose individuals to elevated levels of aggression. In alignment with this, studies have demonstrated that children exhibiting psychopathic tendencies and displaying both aggressive and non-emotional behaviors tend to exhibit higher levels of proactive aggression compared to reactive aggression [27, 28]. Additionally, these children have been found to display a heightened predisposition towards criminality, delinquency, and conduct disorders [19, 29, 30].

A variety of cortical and subcortical regions have been identified as playing a role in the manifestation of aggressive behavior [28]. These include the orbitofrontal cortex (OFC), the ventromedial prefrontal cortex (vmPFC), the dorsolateral prefrontal cortex (dlPFC), and the limbic system. This network is responsible for the processing of emotional and goal-directed behavior, and its damage and/or dysfunction may result in emotional dysregulation, which in turn gives rise to impulsive and aggressive behaviors [31–33].

The prefrontal cortex, which is primarily associated with complex executive functions, inhibitory control, and emotion regulation, through its connections with the limbic system, serves as a subcortical mediator [34] of impulsive and aggressive tendencies [35]. Indeed, reactive aggression represents the consequence of an imbalance between the control of top-down processes derived from the OFC, dlPFC, and anterior cingulate cortex (ACC) and the activation of bottom-up processes initiated by limbic regions, including the amygdala and the insula [36–38]. In contrast, proactive aggression is associated with the activation of the vmPFC and OFC, which are essential for risk assessment and the strategic planning of aggressive actions [39]. Several studies [40, 41] have also demonstrated a comparable effect resulting from a dysfunction of the dlPFC or its connectivity with other brain regions, including the limbic system.

The neural mechanisms underlying these two forms of aggression are different also with respect to hemispheric lateralization, in particular with reference to the frontal asymmetry model [42]; the avoidance or withdrawal-oriented reaction is essentially related to right frontal brain activity, conversely, approach-related motivation with respect to the stimulus is related to left frontal brain activity [43–46]. Indeed, aggressive behavior and mental states associated with anger (i.e., approach-related) have been linked with higher activation of the left prefrontal cortex [47]. In particular, the left dlPFC has been shown to be engaged during aggressive responses, especially in the context of proactive aggression [48–51]. However, some studies have also indicated that a reduction in the functioning of the left dlPFC may be associated with antisocial disorders, including a lack of behavioral control and impulsiveness [52]. Nevertheless, a recent meta-analysis [53] has called into question the prefrontal hypoactivity model in reactive aggression. These findings suggest that both proactive and reactive control may be associated with both left and right dlPFC activity [54, 55].

An asymmetric prefrontal involvement emerges also from non-invasive brain stimulation studies, in particular those using transcranial direct current stimulation (tDCS). Dambacher and colleagues [56] investigated the effect of anodal tDCS over the right dlPFC on aggressive behavior within healthy subjects, showing a significant difference in proactive aggression during active tDCS within male subjects, but not within females. Specifically, men who received active stimulation exhibited a lower level of aggression than those who received sham tDCS [56]. Similarly, Weidler and colleagues [57] employed the same experimental task within a sample of male participants, comprised of alcohol consumers, smokers, and healthy controls. One group received anodal tDCS over the right dlPFC, while the other underwent sham tDCS. Prior to and following the stimulation, participants completed an adapted version of the Taylor Aggression Paradigm – TAP [58]. Only alcohol users, following active stimulation, exhibited a decrease in aggressive behavior [57]. In addition, another recent study, which aimed to enhance empathic abilities and reduce aggressive behavior within a cohort of forensic patients, reported an overall decrease in aggressive responses and self-reported reactive (but not proactive) aggression following active tDCS over the bilateral vmPFC [59].

It is also essential to consider how inter-individual differences, such as sex and personal disposition, may influence the effects of tDCS on aggressive behavior. Previous studies have shown that these differences play a significant role in modulating cortical excitability and the effects of brain stimulation on aggressive tendencies [60, 61]. Indeed, it has been demonstrated that hormones, such as testosterone, can influence the desire to obtain or maintain a higher-status position [62, 63]. Previous studies have corroborated the assertion that a lower-status position is associated with heightened aggression [64]. This phenomenon is particularly relevant in males, who exhibit heightened levels of aggressive behavior when confronted with a potential rival [65]. Other evidence showed alterations in aggressive behavior between male and female participants. During stimulation of the ventrolateral prefrontal cortex (vlPFC), males exhibited heightened aggression relative to females during sham tDCS, whereas females showed comparable levels of aggression to males during active tDCS [66].

In this context, the aim of the present study was to contribute to the investigation of the dissociation of proactive and reactive mechanisms of aggression and their neurobiological substrates by exploring the effects of bilateral stimulation of the dlPFC by tDCS. Participants underwent three stimulation sessions in a double-blind, randomized order: anodal stimulation of the right dlPFC with concurrent cathodal stimulation of the left dlPFC; cathodal stimulation of the right dlPFC with concurrent anodal stimulation of the left dlPFC; sham bi-hemispheric prefrontal stimulation. The behavioral effects within all conditions were evaluated by means of the modified Taylor Aggression Paradigm – mTAP [58, 67], also exploring whether personal dispositions such as levels of aggression, impulsiveness, and empathy are associated with the modulation of proactive and reactive aggressive responses. To our knowledge, this is the first study to assess whether bilateral prefrontal transcranial direct current stimulation (tDCS) differentially affects proactive and reactive aggression. A key strength of the study lies in its within-subjects design, which allowed for direct comparisons between two prefrontal stimulation conditions with opposite polarity (right anodal/left cathodal vs. right cathodal/left anodal), as well as with a sham (placebo) condition. Drawing on previous results [68], we formulated the following hypotheses: with respect to males, we expected an increase in aggressive responses during right anodal/left cathodal tDCS (Hp. 1), and no increase during the opposite stimulation (right cathodal/left anodal) (Hp. 2); with respect to females, we hypothesized either an opposite pattern of neuromodulation or no effect during right anodal/left cathodal tDCS (Hp. 3), but no effect at all with the right cathodal/left anodal stimulation (Hp. 4).

## Methods and materials

### Participants

The sample size estimation was calculated by means of an a priori power analysis (effect size *f* = 0.25; statistical power = 0.8; alpha error level *p* = .05) using the software G*Power 3.1 [69], which showed a recommended sample size of at least 28 participants to achieve enough statistical power.

To face possible dropouts, 30 healthy participants were recruited and took part in the study (15 males and 15 females); they were all white/Caucasian, right-handed, and with normal or corrected-to-normal vision. They all provided their written informed consent prior to the experiment. The study protocol was approved by the Ethical Committee of the University of Milano-Bicocca and in concordance with the Declaration of Helsinki. Demographic characteristics of the sample and scores obtained at the self-report questionnaires are reported in Table 1.

**Table 1:** Demographic characteristics and self-report questionnaires’ scores of the sample

	Males (*N* = 15)	Females (*N* = 15)	t-value	*p*-value
Age (years)	23.93 ± 3.79	23.73 ± 2.74	0.17	0.44
Education (years)	15.67 ± 2.58	16.07 ± 1.83	− 0.49	0.31
BPAQ	69 ± 16.31	64 ± 11.21	0.98	0.17
BIS-11	60.27 ± 8.15	55.67 ± 5.89	1.77	0.04*
IRI	96.13 ± 8.51	99.33 ± 6.3	-1.17	0.13
RPQ-Reactive	9.27 ± 4.53	6.53 ± 3.36	1.88	0.04*
RPQ-Proactive	1.6 ± 1.64	0.53 ± 0.83	2.25	0.02*

All data are reported with mean ± standard deviation. Data were compared by means of independent samples *t*-tests

Abbreviations: *BPAQ* Aggression Questionnaire, *BIS* Barratt Impulsiveness Scale, *IRI* Interpersonal Reactivity Index, *RPQ* Reactive Proactive Aggression Questionnaire

*Significant difference

### Transcranial direct current stimulation (tDCS)

The tDCS was administered with the BrainSTIM stimulator (EMS s.r.l, Bologna, Italy; *emsmedical.net)*. The BrainSTIM stimulator is programmable and allows the management of real or placebo stimulation protocols, which were activated with codes for conducting double-blind studies. All participants were healthy, with exclusion criteria including a history of psychiatric and/or neurological disorders or any contraindications to tDCS, as determined with an ad hoc screening questionnaire [70] (see below for details). No participant reported past or current neurological or psychiatric conditions, personality disorders, or substance use or abuse. The tDCS protocol involved the use of two electrodes (anode and cathode), each of 35 cm^2^; after being placed in two saline-soaked sponges, accordingly to the experimental session, one electrode (anode or cathode) was placed on the scalp over the right or left dlPFC (F3 or F4), while the other electrode was placed over the contralateral dlPFC, following the international EEG 10–20 system for electrodes placements. The real stimulation consisted of the continuous application of a current at an intensity of 1.5 mA for 20 min (fade-in/fade-out phases = 30 s). The same stimulation was used for sham tDCS, but the stimulator was turned off after 30 s to ensure that participants could feel the initial sensations at the beginning of the stimulation, a requisite for successful masking [70]. The tDCS device was set in advance to deliver real or sham tDCS, thus keeping both the participant and the experimenter blinded to tDCS allocation. At the end of each tDCS session, participants were administered a 7-item questionnaire [71] to assess potential side effects of the stimulation. No serious adverse events were reported. Only mild and transient side effects (e.g., itching, dizziness, and tingling) were described by a limited number of participants (i.e., 23% of the sample), and none reported any interference with their task performance (see also: [72, 73]). Only one out of 30 participants (i.e., 3% of the sample) was able to correctly guess all stimulation conditions, indicating an effective blinding procedure.

### Modified Taylor Aggression Paradigm (mTAP)

**Fig. 1:**
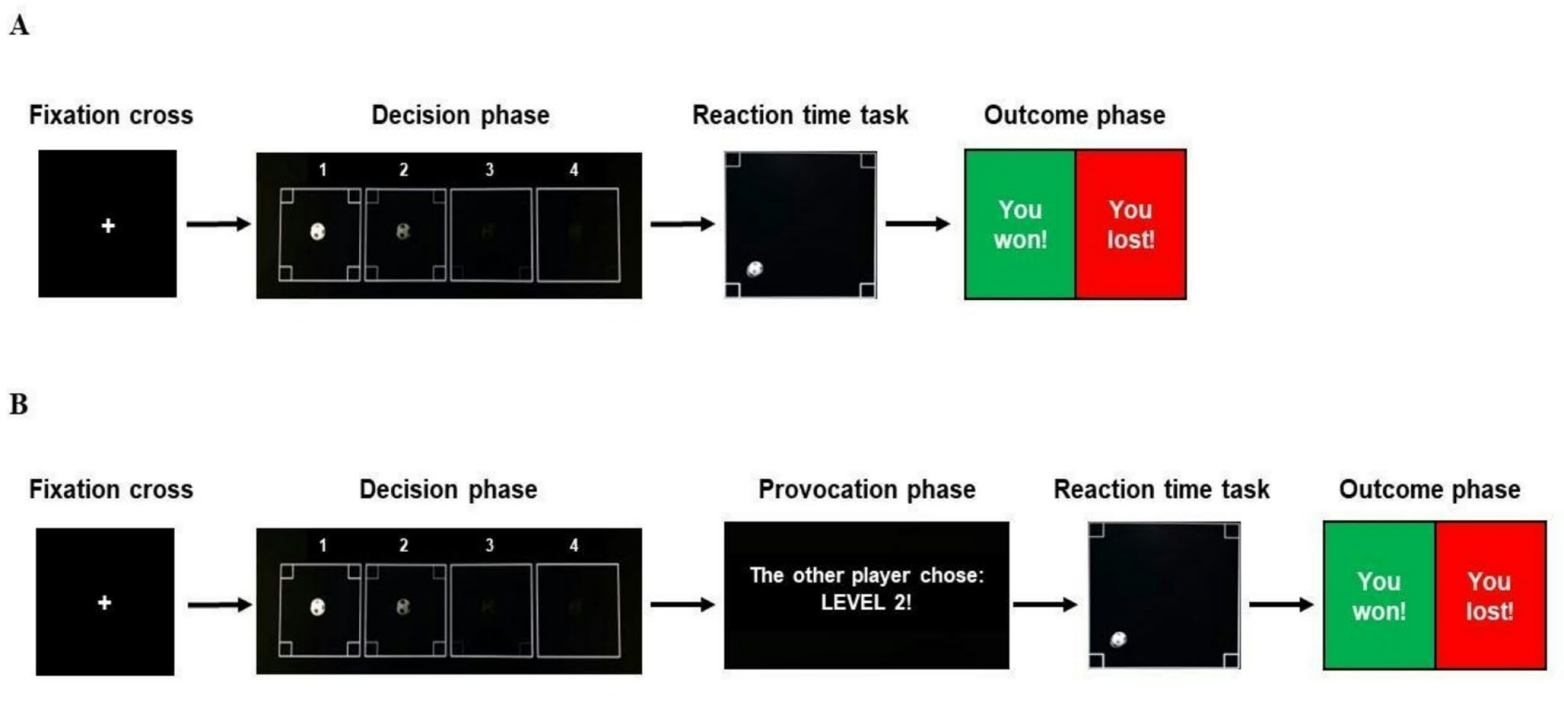
Visual representation of one trial of the proactive (**A**) and reactive (**B**) Taylor Aggression Paradigm

The Taylor Aggression Paradigm [58] is one of the most widely used tasks in aggression research; it consists of a series of reaction time tasks played by the participant against an alleged opponent. Before each trial, both players must choose a punishment level for the opponent, which the player who loses the game will receive. The modified versions of this task include two versions, one measuring proactive aggression (pTAP) and the other measuring reactive aggression (rTAP) [67]. First, participants look at a fixation cross for a randomized interval of time, within a range from 1 to 3 s. Afterward, in both the pTAP and the rTAP, the task consists of pressing the space bar on the keyboard as soon as the ball displayed on the monitor reaches any of the four corners at the borders of the screen (see Fig. 1). As with every reaction time task, participants are told that they must be faster than their opponent in order to win the game. Importantly, players have to choose the level of interference for their opponent (i.e., the blurriness applied to the screen of the opponent) before the start of the task—in a so-called decision phase. Level 1 implies normal screen visibility, whereas levels 2, 3, and 4 indicate a progressive increase in the opponent’s screen level of blurriness. During the decision phase, examples of how the screen of the opponent would look like in the four different blurriness conditions are made available on the monitor. After each trial participants see if they won or lost as indicated by a green or red screen, respectively, in a so-called outcome phase. The task is preprogrammed so that each level of chosen interference is associated with a certain probability of winning the trial. Specifically, level one corresponds to a 30% chance of winning the trial, level two to a 50% chance, level three to a 70% chance, and level four to a 90% chance. Participants play a total of 40 trials for each of the two tasks, for a duration of approximately 5 min for the pTAP and 10 min for the rTAP. However, there is one fundamental difference between them: in the rTAP, prior to the beginning of each trial, the decision of the opposing player regarding the level of blurriness is displayed on the screen, so that the participant can see it (provocation phase); on the contrary, the pTAP is deprived of this provocative element, as it is designed to ascertain the deliberate and provoked deployment of aggressive behavior, as represented by the levels of blurriness. Moreover, the rTAP provocation level (i.e., the level of blurriness of the screen chosen by the opponent) is preprogrammed to gradually increase during the task. Initially, participants are mostly unprovoked or provoked at a low level; nonetheless, every ten trials, the frequency of no and low provocation trials (levels 1 and 2) decreases while that of medium and high provocation trials (levels 3 and 4) increases. Each level of chosen interference is associated with a certain probability of winning the trial—as in the pTAP—but adjusted for the level of provocation selected by the opponent, so to make the game more believable. A visual representation of the paradigm is available in Fig. 1.

### Buss and Perry’s Aggression Questionnaire (BPAQ)

The BPAQ [74, 75] is a 29-item questionnaire widely used as a measure of self-reported aggression. Participants are asked to rate each item on a scale from 1 (extremely not characteristic of me) to 5 (extremely characteristic of me). The total score ranges from 29 to 145, with higher scores representing higher levels of aggression. Moreover, four fundamental components of aggression can be derived by subscores: physical aggression (score range = 9–45), verbal aggression (range = 5–25), anger (range = 7–35), and hostility (range = 8–40).

### Reactive-Proactive Aggression Questionnaire (RPQ)

The RPQ [76, 77] was developed with the intent to measure these two dimensions of aggression—reactive and proactive—independently and in physical or verbal forms. It consists of 23 items (12 proactive, 11 reactive) and each item can be rated as 0 (never), 1 (sometimes), or 2 (often) for frequency of occurrence.

### Barratt Impulsiveness Scale (BIS-11)

The BIS-11 represents one of the best-known and most widely used instruments for measuring impulsivity [78, 79]. The BIS-11 is a scale that tends to synthesize the concept of impulsivity around three basic elements: (1) motor impulsiveness (range = 11–44), defined as the tendency to take immediate action as a somehow ‘reflex’; (2) attentional impulsiveness (range = 8–32), an expression of the tendency to initiate actions that are always new and different due to a difficulty to concentrate or focus attention and to easy distractibility; (3) non-planning impulsiveness (range = 11–44), understood as the tendency to make decisions that are not thoughtful with respect to the short-, medium- and long-term consequences of behaviors. The scale BIS-11 consists of 30 items, with each item rated on a 4-point scale (1 = extremely uncharacteristic of me, to 5 = extremely characteristic of me), for a total score ranging from 30 to 120: the greater the score, the greater the impulsivity.

### Interpersonal Reactivity Index (IRI)

The IRI [80, 81] is one of the most commonly used self-report questionnaires for measuring empathy in adults. It consists of 28 items (total score range = 28–140), divided into four sub-scales: fantasy (range = 7–35), perspective taking (range = 7–35), empathic concern (range = 7–35), and personal distress (range = 7–35). Each item is rated on a 4-level scale, from 1 (does not describe me at all) to 5 (describes me perfectly), consequently higher scores reflect higher levels of emphatic abilities.

## Experimental design

A double-blind within-subjects design was used. Every participant underwent three tDCS sessions, separated by a minimum of 3 to a maximum of 10 days, during which they received in a randomized order real (right anodal/left cathodal; right cathodal/left anodal) or sham tDCS.

**Fig. 2:**
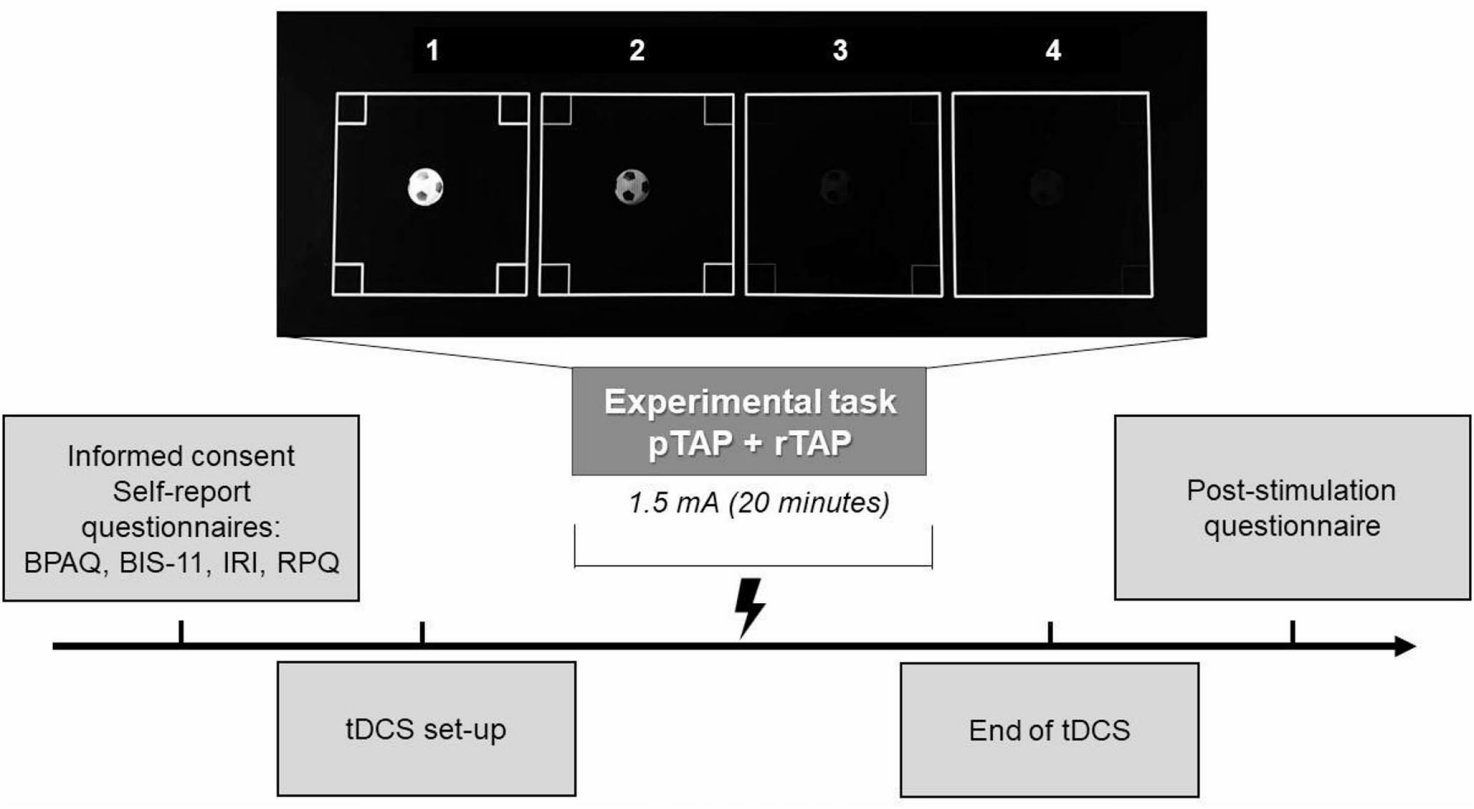
Experimental procedure. Procedure and timeline of the study. The experimental task is here represented during the decision phase

Before starting the first tDCS session, all participants completed the self-report questionnaires in the following order: BPAQ, BIS-11, IRI, and RPQ. Afterward, they underwent the mTAP (both the pTAP and the rTAP, in a randomized order) while receiving tDCS. The stimulation started 5 min before the task began and was delivered during the entire course of the mTAP, which lasted 15 min overall. In one session, the anodal stimulation was delivered over the right dlPFC, and the cathodal stimulation over the left dlPFC (right anodal/left cathodal). In the other session, participants received the opposite stimulation, with the anode positioned over the left dlPFC and the cathode over the right dlPFC (right cathodal/left anodal). At the end of both tDCS sessions, every participant completed the 7-item questionnaire to assess the tDCS side effects. The experimental procedure is illustrated in Fig. 2.

### Data analysis

Analyses of participants’ responses were performed by means of IBM SPSS Statistics, version 28.0 [82] and R Studio, version 4.2.3 [83].

Descriptive statistics are reported in Table 1, along with comparisons by means of independent samples *t*-tests to verify differences between males and females with respect to age, education, and scores at the self-report questionnaires.

Three trials from participant 9 had to be excluded due to an initial error in the execution of the task; hence, since the number of measurements was not equal across participants, a mixed-effects model was used for the analyses. Specifically, as previously done by Boccadoro et al. [67], a trial-by-trial analysis was performed by fitting a linear mixed-effects model including random intercepts for participants, so as to account for repeated measures.

Two separate analyses were conducted for proactive and reactive aggression (pTAP and rTAP, respectively). Sex (male vs. female) was entered in the model as the between-subjects factor, while tDCS (right anodal/left cathodal vs. right cathodal/left anodal vs. sham) as the within-subjects factor. The chosen level of blurriness of the screen (i.e., the aggression choice) for each trial from the second trial onward (either proactive or reactive) was entered as the dependent variable.

Lastly, correlations between the pTAP and rTAP responses and scores at the self-report questionnaires (BPAQ, BIS-11, IRI, RPQ) were conducted using the Benjamini-Hochberg correction to adjust for multiple comparisons.

## Results

### Linear mixed-effects model analyses

**Fig. 3:**
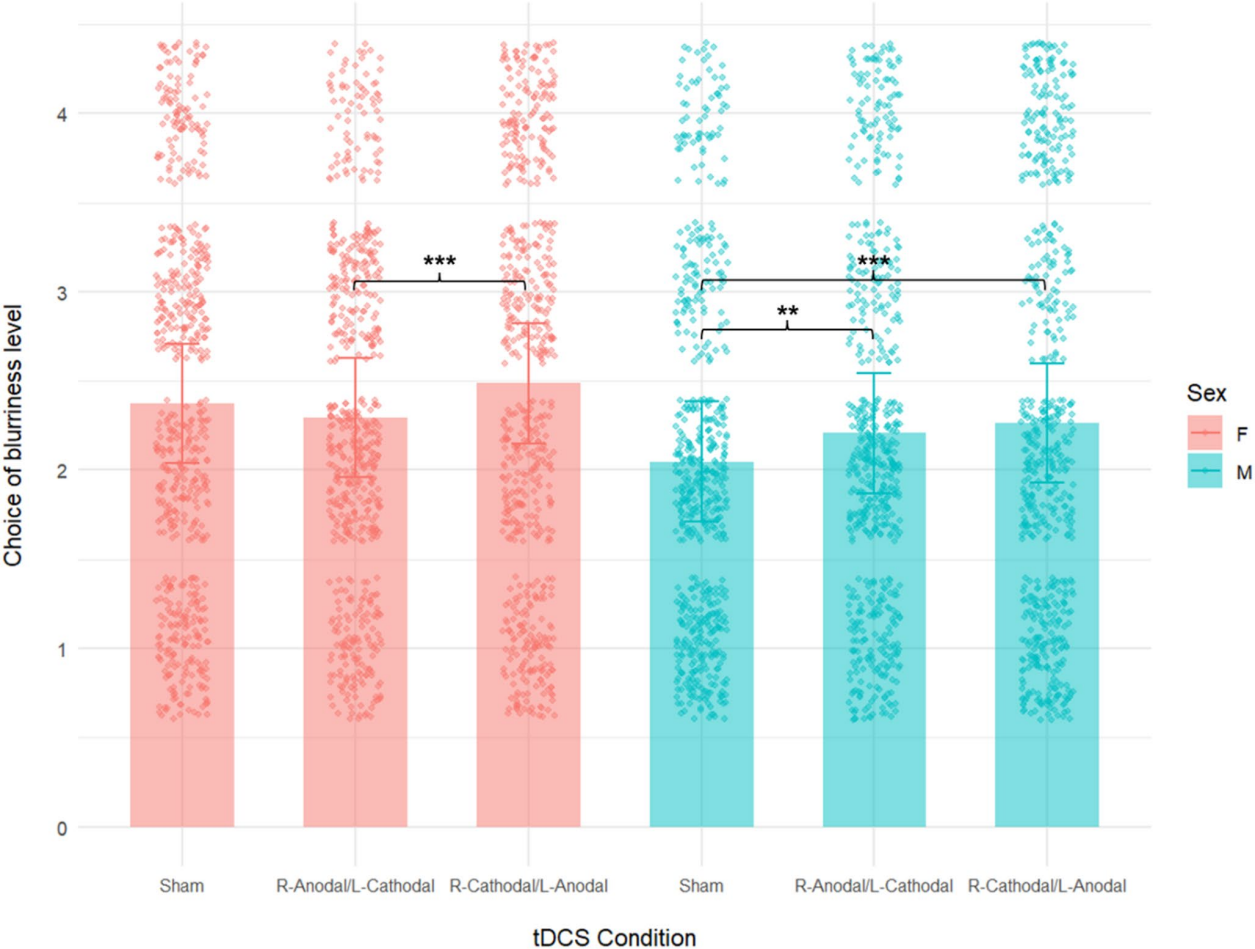
Results of the linear mixed-effects model for the proactive Taylor Aggression Paradigm (pTAP). The figure shows the chosen level of blurriness of the screen (i.e., aggression choice) of males and females across stimulation conditions (sham; right anodal/left cathodal; right cathodal/left anodal). Raw individual responses are overlaid. F = females; M = males; error bars = 95% confidence intervals; ***=*p* < .001; **=*p* < .01.

With regard to the pTAP, the analyses reported a significant main effect of right cathodal/left anodal tDCS, as well as a significant interaction between Sex and tDCS for the right anodal/left cathodal stimulation (see Table 2; Fig. 3). During right cathodal/left anodal tDCS, participants showed higher levels of aggression, as indexed by their choices (i.e., blurriness level of the screen). This main effect was found regardless of sex, as it is shown in Fig. 3. The significant interaction between Sex and tDCS—which was found exclusively for the anodal condition—was further investigated by means of Bonferroni-corrected post-hoc comparisons.

**Table 2:** Fixed effects estimates from the linear mixed-effects model analyses for aggression choices in the proactive Taylor Aggression Paradigm (pTAP)

Predictor	Estimate	SE	t	*p*
Intercept	2.28	0.12	18.41	< 0.001
Sex	− 0.22	0.25	− 0.88	0.39
tDCS (right anodal/left cathodal)	0.04	0.03	1.32	0.19
tDCS (right cathodal/left anodal)	**0.16**	**0.03**	**4.9**	**< 0.001**
Sex X tDCS (right anodal/left cathodal)	**0.24**	**0.07**	**3.54**	**< 0.001**
Sex X tDCS (right cathodal/left anodal)	0.1	0.07	1.56	0.12

Abbreviations: *SE* Standard Error, *tDCS* transcranial direct current stimulation

Bold values denote statistical significance at the *p *< 0.05 level

The significant interaction between Sex and tDCS—which was found exclusively for the right anodal/left cathodal condition—was further investigated by means of Bonferroni-corrected post-hoc comparisons. During right anodal/left cathodal stimulation, males selected significantly higher aggressive responses as compared to sham tDCS (2.21 vs. 2.05), while females showed the opposite behavioral trend (2.29 vs. 2.37), although it did not reach statistical significance (see Fig. 3).^1^

**Fig. 4:**
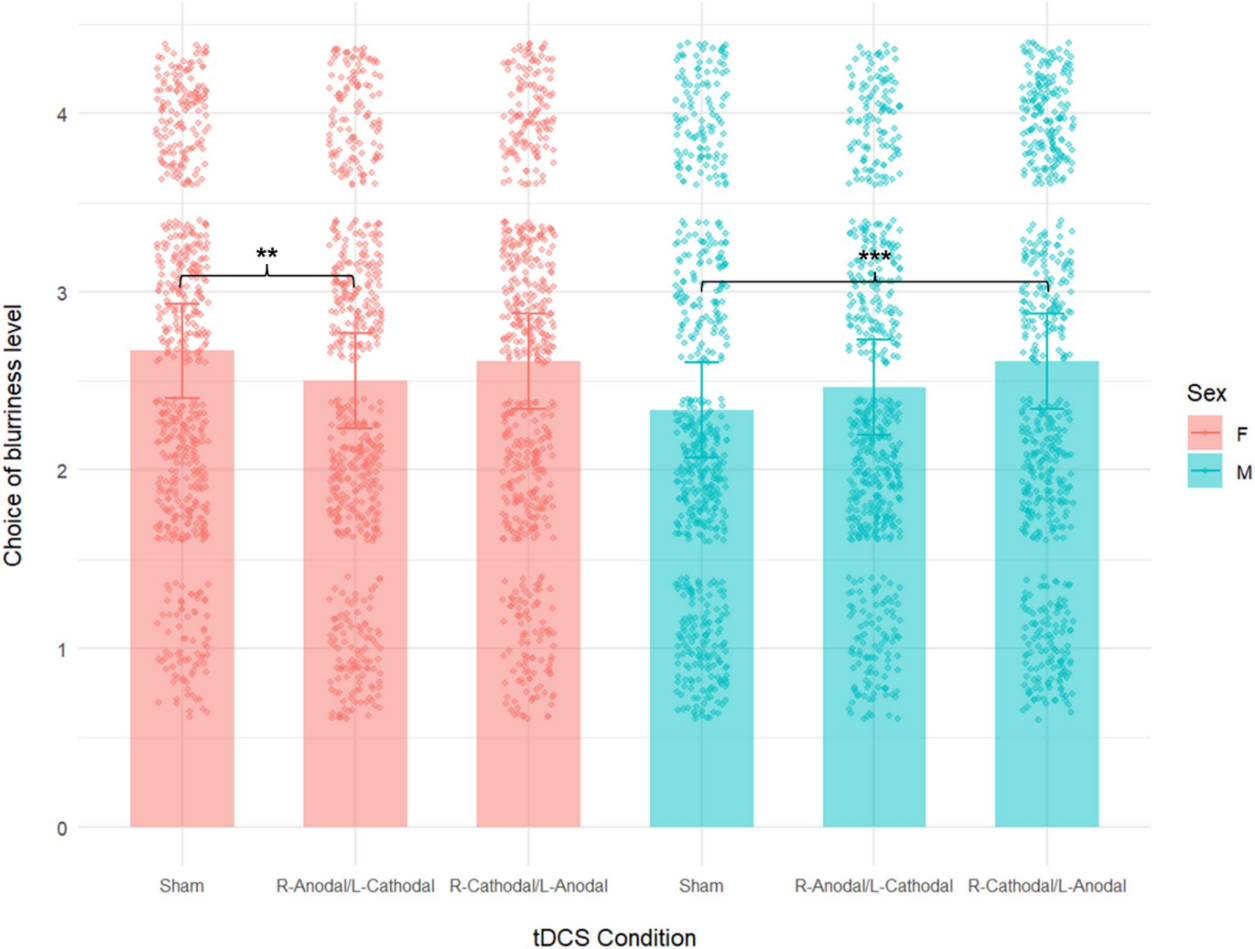
Results of the linear mixed-effects model for the reactive Taylor Aggression Paradigm (rTAP). The figure shows the chosen level of blurriness of the screen (i.e., aggression choice) of males and females across stimulation conditions (sham; right anodal/left cathodal; right cathodal/left anodal). Raw individual responses are overlaid. F = females; M = males; error bars = 95% confidence intervals; ***=*p* < .001; **=*p* < .01.

With regard to the reactive aggression (i.e., the rTAP), the analyses showed a significant main effect of right cathodal/left anodal tDCS, as well as a significant interaction between Sex and tDCS for both the right anodal/left cathodal and right cathodal/left anodal stimulation (see Table 3; Fig. 4).

**Table 3:** Fixed-effects estimates from the linear mixed-effects model analyses for aggression choices in the reactive Taylor Aggression Paradigm (rTAP)

Predictor	Estimate	SE	t	*p*
Intercept	2.53	0.1	25.99	< 0.001
Sex	− 0.12	0.19	− 0.63	0.54
tDCS (right anodal/left cathodal)	− 0.02	0.04	− 0.65	0.52
tDCS (right cathodal/left anodal)	**0.11**	**0.04**	**2.89**	**0.004**
Sex X tDCS (right anodal/left cathodal)	**0.3**	**0.07**	**4.14**	**< 0.001**
Sex X tDCS (right cathodal/left anodal)	**0.34**	**0.07**	**4.68**	**< 0.001**

Abbreviations: *SE* Standard Error, *tDCS* transcranial direct current stimulation

Bold values denote statistical significance at the *p *< 0.05 level

For reactive aggression as well, during right cathodal/left anodal tDCS participants selected higher levels of aggression, as shown in Fig. 4.

However, in this case, only males showed a significant difference between sham and right cathodal/left anodal tDCS (2.34 vs. 2.61), while females did not (2.67 vs. 2.61).

On the other hand, females displayed a significant difference between the level of aggression choices during right anodal/left cathodal stimulation compared to sham tDCS; specifically, their aggressive responses were significantly reduced during the active stimulation (2.5 vs. 2.67), as can be seen in Fig. 4.^2^

### Correlations

The correlation analyses showed a positive association between the mean level of aggression choices at the pTAP (i.e., mean level of blurriness of the screen without provocation) and the BIS-11 total score in males only (*ρ* = 0.54, *p* = .004). In particular, a significant correlation between the mean level of proactive aggressive responses and the score at the attentional impulsiveness sub-scale was reported (*ρ* = 0.63, *p* = .001). Moreover, males showed a positive association between the mean level of aggression choices at the pTAP and the total score at the BPAQ (*ρ* = 0.45, *p* = .006), as well as with the hostility sub-score (*ρ* = 0.48, *p* = .004). A significant correlation between the level of proactive aggressive responses and the score at the personal distress sub-scale of the IRI was also reported in males (*ρ* = 0.65, *p* < .001). No significant correlation was found in females (all *p*_*s*_ ≥ 0.4).

Regarding the rTAP, a significant correlation was found between the mean level of reactive aggressive responses (i.e., mean level of blurriness of the screen with previous provocation) and the score obtained at the RPQ reactive sub-scale in males (*ρ* = 0.48, *p* = .005); moreover, males showed a positive association between the BIS-11 total score and reactive aggression (*ρ* = 0.42, *p* = .012). In particular, for reactive aggression as well, significant correlations between the mean level of aggression choices and the score at the attentional impulsiveness (*ρ* = 0.57, *p* = .005) and motor impulsiveness (*ρ* = 0.36, *p* = .03) sub-scales were reported. Moreover, males showed a positive association between the mean level of aggression choices at the rTAP and the total score at the BPAQ (*ρ* = 0.38, *p* = .028), as well as with the hostility sub-score (*ρ* = 0.45, *p* = .008). A significant correlation between the level of reactive aggressive responses and the score at the personal distress sub-scale of the IRI was also reported in males (*ρ* = 0.49, *p* = .005). In females, no significant correlation was observed (all *p*_*s*_ ≥ 0.35). All correlations are reported in Table 4.

**Table 4:** Correlation analyses between the mean level of blurriness (i.e., mean level of aggression choices) and the scores obtained at the self-report questionnaires in males and females

pTAP	Males		Females	
	Spearman’s ρ	*p*-value	Spearman’s ρ	*p*-value
*BPAQ*	0.449	0.006**	0.139	0.495
Physical aggression	0.150	0.375	− 0.006	0.997
Verbal aggression	0.243	0.147	− 0.001	0.997
Anger	0.375	0.028*	0.176	0.422
Hostility	0.483	0.004**	0.215	0.422
*BIS-11*	0.540	0.004**	− 0.217	0.422
Attentional impulsiveness	0.632	0.001***	− 0.191	0.422
Motor impulsiveness	0.281	0.102	− 0.174	0.422
Non-planning impulsiveness	0.248	0.147	− 0.100	0.641
*IRI*	0.320	0.069	0.193	0.422
Perspective taking	− 0.205	0.220	− 0.187	0.422
Fantasy scale	0.122	0.456	0.177	0.422
Empathic concern	0.077	0.615	0.222	0.422
Personal distress	0.649	0.001***	− 0.032	0.963
*RPQ-Proactive*	0.292	0.098	− 0.143	0.495

rTAP	Spearman’s ρ	*p*-value	Spearman’s ρ	*p*-value
*BPAQ*	0.377*	0.028*	0.052	0.920
Physical aggression	0.172	0.352	0.021	0.956
Verbal aggression	0.158	0.375	− 0.137	0.522
Anger	0.275	0.113	0.098	0.852
Hostility	0.446	0.008**	0.154	0.673
*BIS-11*	0.424	0.012*	− 0.204	0.537
Attentional impulsiveness	0.567	0.005**	− 0.224	0.522
Motor impulsiveness	0.361	0.032*	− 0.239	0.522
Non-planning impulsiveness	0.074	0.725	− 0.029	0.956
*IRI*	0.181	0.351	0.087	0.852
Perspective taking	− 0.289	0.101	− 0.106	0.852
Fantasy scale	0.054	0.778	− 0.070	0.884
Empathic concern	− 0.017	0.910	0.299	0.522
Personal distress	0.488	0.005**	− 0.001	0.995
*RPQ-Reactive*	0.482	0.005**	− 0.166	0.673

Abbreviations: BPAQ = Buss & Perry Aggression Questionnaire; BIS = Barratt Impulsiveness Scale; IRI = Interpersonal Reactivity Index; RPQ = Reactive-Proactive Aggression Questionnaire. ***= *p* < 0.001; **= *p* < 0.01; *= *p* < 0.05

## Discussion

The present study examined whether and how asymmetrical modulation of prefrontal excitability—via right anodal/left cathodal and right cathodal/left anodal tDCS—affects proactive and reactive aggression. The primary aim was to clarify the role of the dlPFC in these distinct forms of aggressive behavior and to determine whether the effects of neuromodulation vary based on the stimulated hemisphere and aggression type. Moreover, in light of previous findings, the study explored the influence of sex on tDCS effects, together with their association with individual dispositional traits, namely aggressiveness, impulsivity, and empathy.

Results show that proactive aggressive responses in males increase during right anodal/left cathodal stimulation; however, this effect is even more consistent for the opposite stimulation, namely right cathodal/left anodal tDCS. Interestingly, females show an increase in proactive aggression choices during right cathodal/left anodal tDCS, as compared to right anodal/left cathodal stimulation but not with respect to sham tDCS.

With regard to reactive aggressive behavior (i.e., the rTAP), males show an increase in aggression choices during right cathodal/left anodal tDCS, as encountered with proactive aggression as well. On the other hand, anodal stimulation reduces reactive aggression only in females.

Overall, these findings show that tDCS applied over the dlPFC can modulate proactive and reactive aggressive responses in both sexes, increasing them mainly through right cathodal stimulation with concurrent left anodal stimulation, in line with the motivational direction model of frontal asymmetry, according to which avoidance/withdrawal from the stimulus would be associated with hypoactivation of the right PFC, whereas approaching the angry stimulus would be associated with hyperactivation of the left PFC [42, 46, 84]. It seems plausible to suggest that trans-callosal inhibition, which is increased by the application of right anodal/left cathodal tDCS on the dlPFC, may further inhibit the left dlPFC, which has already been targeted by cathodal stimulation. This, therefore, results in the promotion of a dysfunctional prefrontal inhibitory neurotransmission. Indeed, decreased activation of the left prefrontal cortex has been demonstrated to result in deficits in impulsivity, response inhibition, antisocial behavior, and lack of behavioral control [52, 85]. However, males’ proactive aggression appears to be modulated also by applying the anode over the right dlPFC and the cathode over the left homologous area, indicating a more widespread activation pattern in the emergence of proactive aggressive behavior, as compared to females.

This evidence seems to underline that, while females tend to act in accordance with the motivational direction model of frontal asymmetry [42], males have a less enclosed activation pattern, possibly related to an overall interhemispheric imbalance between the left and right dlPFC, in absence of a specific direction. This assumption is in line with the meta-analysis from Yang and Raine [52]—conducted on an antisocial sample comprised of 83.9% percentage of males across studies—which reported a widespread decrease in prefrontal functioning in the right OFC, left dlPFC, and right ACC.

A recent study has reported that—during young adulthood—women exhibit more cortical lateralization than men, showing significantly smaller cortical complexity of cerebral folding in the left frontal, parietal, and occipital lobes and in the right temporal lobe [86]. In line with these findings, an interesting—though not statistically significant—trend can be observed in the present study, which may suggest a more lateralized pattern in the female brain. Indeed, in females, proactive aggression shows a tendency to increase during right cathodal/left anodal tDCS—mirroring the pattern observed in males—whereas reactive aggression tends to decrease during right anodal/left cathodal stimulation. These results suggest that reactive and proactive aggression partially rely on distinct neural substrates, which appear to be sensitive to sex-related differences. Moreover, they may point to a more specific involvement of the right dlPFC in the regulation of aggressive behavior within females. This interpretation aligns with a recent fMRI study by Repple and colleagues [87], which investigated sex differences in the neural correlates of reactive aggression in a simulated social context. Although males and females did not differ significantly in their level of aggressive responses, distinct patterns of brain activation were observed. In males, aggression was positively correlated with activity in the left amygdala, orbitofrontal cortex (OFC), and anterior cingulate cortex (ACC), whereas in females, activation in these regions was negatively correlated with aggression, suggesting a potential inhibitory role. These findings indicate that while males and females engage a common neural network for aggression, they may rely on different emotion regulation strategies, which could differentially influence the control of aggressive behavior. As suggested by a review by Mancke et al. [88], male and female patients with borderline personality disorder—characterized by dysregulated anger and behavioral manifestations—seem to have a different pattern of neurobiological alterations. Indeed, only males show more extended volume reductions in the orbital frontal cortex and the ventromedial prefrontal cortex [89] as well as in the superior, medial and middle frontal gyrus [90], while specific alterations in female patients are rare.

It is worth noting, as can be observed in Table 1, that males report a higher level of aggression—both in its reactive and proactive form—than females, which is in line with prior literature [91, 92]. Moreover, they report higher levels of impulsivity at the BIS-11, a measure highly correlated with forms of reactive aggression, especially in males [91, 93]. Indeed, there is a significant association between the level of aggressive responses—both at the pTAP and the rTAP—and self-reported impulsiveness in male participants. A possible explanation is that—in order to compensate for a lack of focus on the task at hand—males with higher impulsivity traits decide to choose more aggressive responses so as to have a higher chance of winning against the opponent. Among the BIS-11 sub-scales, attentional impulsiveness appears to cover a specific role in the emergence of both proactive and reactive aggressive behavior in males. A study by Martin and colleagues [94] has reported how, in a sample of detainees with antisocial personality disorder, attentional impulsivity was significantly related to the recidivism rate. Moreover, attentional impulsiveness was found to explain unique variance in physical aggression, anger, and hostility in a sample of male violent offenders [95]. Indeed, the total score at the BPAQ—and hostility in particular—showed a significant association with the level of aggressive responses both at the pTAP and the rTAP in male participants. Another significant correlation was found between the reactive RPQ score and the mean level of aggression choices during the rTAP, which is consistent with the construction of the RPQ itself [76]. Moreover, the level of personal distress at the IRI showed a significant correlation with aggression choices at the pTAP and the rTAP; indeed, affective/self-oriented empathy—which includes personal distress—has been shown to be positively related to reactive aggression [96], pointing to the importance of considering the multidimensional character of empathy and aggression itself.

There are some limitations to the study that should be here discussed. First, our sample was comprised of healthy participants, who were all young adults and of white/Caucasian ethnicity. It has been observed that risk factors for aggression vary according to both age [97] and racial/ethnic differences [98], as well as sex. Therefore, in the future, it might be useful to investigate more heterogeneous samples—by also considering sociodemographic variables such as age and ethnicity—in order to generalize the findings to broader populations. Secondly, it is essential to examine the composition of the mTAP. This paradigm proves to be a valid tool for distinguishing between reactive and proactive aggression; the pTAP is constructed on the basis of an instrumental motivation (winning the game), which allows the relevant intentional act to be measured in the absence of provocation. In contrast, the rTAP elicits a more impulsive/reactive aggression based on the opponent’s provocation. Nonetheless, loss outcomes within the pTAP might introduce an element of frustration during the task, which could contain a potential reactive aggressive component as well as a proactive one. Consequently, proactive and reactive aggression elements in this task cannot be completely separated [67]. However, this limitation reflects what happens in real-life scenarios as well, where aggressive behaviors are usually not related to a single motivation but rather entail a combination of different motivations, including anger, revenge, reward, and recreation [99].

In conclusion, the present study demonstrates that bi-hemispheric tDCS over the dlPFC can significantly modulate aggression and—at least partially—dissociate between proactive and reactive aggression. Moreover, it suggests that males and females have different activation patterns in relation to the emergence of aggressive behavior, which can be differentially modulated by means of non-invasive brain stimulation. Personality traits—in particular, attentional impulsiveness and hostility—seem to be also related to the level of aggressive behavior displayed; however, this association appears to be independent of the polarity of the stimulation. These results demonstrate the necessity for the development and enhancement of effective instruments for the investigation of behavioral and physiological correlates of aggression and corroborate once again the relevance of taking into account sex and individual differences, which play an integral role in the modulation of cortical excitability and subsequently influence the outcome of tDCS on behavioral manifestations [60, 61, 100].

## Data Availability

The study was preregistered on OSF as 10.17605/OSF.IO/5TW8K, and data are available at *osf.io/4w8eh*.

## Footnotes

1 The main findings were consistent regardless of whether *z-*scores of trait measures were included as covariates.

2 Including *z-*scores of trait measures as covariates did not affect the results.
